# *Solanum lycopersicum* GOLDEN 2-LIKE 2 transcription factor affects fruit quality in a light- and auxin-dependent manner

**DOI:** 10.1371/journal.pone.0212224

**Published:** 2019-02-12

**Authors:** Alessandra Cavalcanti Duarte Lupi, Bruno Silvestre Lira, Giovanna Gramegna, Bruna Trench, Frederico Rocha Rodrigues Alves, Diego Demarco, Lazaro Eustáquio Pereira Peres, Eduardo Purgatto, Luciano Freschi, Magdalena Rossi

**Affiliations:** 1 Departamento de Botânica, Instituto de Biociências, Universidade de São Paulo, São Paulo, SP, Brazil; 2 Departamento de Ciências Biológicas, Escola Superior de Agricultura ¨Luiz de Queiroz¨, Universidade de São Paulo, Piracicaba, SP, Brazil; 3 Departamento de Alimentos e Nutrição Experimental, Faculdade de Ciências Farmacêuticas, Universidade de São Paulo, São Paulo, SP, Brazil; University of Tsukuba, JAPAN

## Abstract

Plastids are organelles responsible for essential aspects of plant development, including carbon fixation and synthesis of several secondary metabolites. Chloroplast differentiation and activity are highly regulated by light, and several proteins involved in these processes have been characterised. Such is the case of the GOLDEN 2-LIKE (GLK) transcription factors, which induces the expression of genes related to chloroplast differentiation and photosynthesis. The tomato (Solanum lycopersicum) genome harbours two copies of this gene, SlGLK1 and SlGLK2, each with distinct expression patterns. While the former predominates in leaves, the latter is mainly expressed in fruits, precisely at the pedicel region. During tomato domestication, the selection of fruits with uniform ripening fixed the mutation Slglk2, nowadays present in most cultivated varieties, what penalised fruit metabolic composition. In this study, we investigated how SlGLK2 is regulated by light, auxin and cytokinin and determined the effect of SlGLK2 on tocopherol (vitamin E) and sugar metabolism, which are components of the fruit nutritional and industrial quality. To achieve this, transcriptional profiling and biochemical analysis were performed throughout fruit development and ripening from SlGLK2, Slglk2, SlGLK2-overexpressing genotypes, as well as from phytochrome and hormonal deficient mutants. The results revealed that SlGLK2 expression is regulated by phytochrome-mediated light perception, yet this gene can induce chloroplast differentiation even in a phytochrome-independent manner. Moreover, auxin was found to be a negative regulator of SlGLK2 expression, while SlGLK2 enhances cytokinin responsiveness. Additionally, SlGLK2 enhanced chlorophyll content in immature green fruits, leading to an increment in tocopherol level in ripe fruits. Finally, SlGLK2 overexpression resulted in higher total soluble solid content, possibly by the regulation of sugar metabolism enzyme-encoding genes. The results obtained here shed light on the regulatory network that interconnects SlGLK2, phytohormones and light signal, promoting the plastidial activity and consequently, influencing the quality of tomato fruit.

## Introduction

Plastids are organelles with a great diversity of shapes and functions that are found in all photosynthetic eukaryotes. Besides photosynthesis, these organelles are responsible for diverse functions, such as synthesis and storage of some secondary metabolites [1].

Proplastids are found in meristematic regions of the plant and are the precursor of the other plastid types, whose differentiation is tightly regulated by endogenous and exogenous signals. Several hormones have been described to play a role in the control of chloroplast biogenesis, such as auxins and cytokinins. While the first is known to have an inhibitory role in chloroplast differentiation in roots, the latter stimulates this process [2, 3, 4]. In contrast, in tomato (*Solanum lycopersicum*) fruits, auxins promote chloroplast biogenesis, as evidenced by the increment in the abundance of fully developed chloroplasts when the auxin response repressor *SlARF4* (*AUXIN RESPONSIVE FACTOR 4*) is downregulated [5, 6].

Among the exogenous signals, light is paramount as it ensures that chloroplast differentiation only occurs in appropriate conditions for photosynthetic activity. The red/far-red photoreceptors PHYTOCHROMES (PHYs) play a significant role in this process. In the darkness, PHYs are inactive in the cytoplasm, and upon red light exposure an isomeric alteration of the chromophore leads to a rearrangement of the apoprotein structure that exposes the nuclear signalling domain, leading to their translocation into the nucleus [7]. In the nucleus, PHYs promote the degradation of PHYTOCHROME INTERACTING FACTORS (PIFs), which repress, among other positive regulators of photomorphogenesis, the expression of ELONGATED HYPOCOTYL 5 (HY5) and GOLDEN 2-LIKE (GLKs) transcription factors [8]. GLKs are essential for chloroplast differentiation and maintenance [9, 10] and in *Arabidopsis thaliana* are positively regulated by HY5 [11].

As in most plants, tomato genome harbours two *GLK* copies, *SlGLK1* and *SlGLK2*. While *SlGLK1* is mostly expressed in cotyledons, sepals and leaves, *SlGLK2* is predominantly expressed in fruits, more specifically at the pedicellar portion, originating the so-called green shoulder phenotype [12]. This phenotype was lost along tomato domestication by the fixation of a non-functional truncated SlGLK2 coding allele (*Slglk2*), resulting in a uniform ripening fruit, which facilitates harvesting but at the cost of nutritional quality as ripe fruits contain less sugars [12, 13]. In agreement, *SlGLK2* overexpression along the entire longitudinal axis of the fruit in the mutant background has been shown to promote both sugar and carotenoid metabolism in tomato fruits [12, 13]. Thus, the proper development of fruit chloroplast impacts nutritional quality, by affecting the content of not only photoassimilates but also of secondary metabolites. Another plastid-synthesised family of compounds with important nutraceutical value for human health, yet less studied, are the tocopherols [14,15, 16, 17], for which the consequences of SlGLK2 loss of function in tomato fruit remains unexplored.

Tocopherols occur in four forms (α, β, γ and δ) and are important antioxidant molecules that protect photosynthetic machinery by scavenging singlet oxygen and inhibiting the propagation of lipid peroxidation in thylakoid membranes [18, 19, 20, 21]. In mammals, tocopherols have vitamin E activity, in particular the α form, which is the most abundant in most vegetable organs [22, 23]. Tocopherols are synthesised by the condensation of homogentisate and phytyl diphosphate, products of the shikimate and methylerythritol phosphate pathways, respectively. In tomato, many lines of evidence intertwine the metabolism of chlorophyll and tocopherol, especially along fruit ripening, when the chlorophyll degradation-derived phytol can supply tocopherol biosynthesis as the methylerythritol phosphate pathway products are channeled towards carotenoid biosynthesis [24, 25, 26, 27, 28]. Due to its high consumption, tomato is an important source of tocopherol in the human diet [29].

In this sense, by promoting chloroplast differentiation, GLKs directly affect the nutritional quality of edible crops, such as tomato fruit. However, many aspects of GLK regulation and effects over the metabolism of important nutraceutical compounds remain elusive. To fill this gap, the transcriptional profile of *SlGLK2* and the tocopherol and sugar contents were addressed in tomato fruits from wild-type (*i*.*e*. *SlGLK2*), *Slglk2*, PHY-deficient *aurea* (*au-SlGLK2*) and *au-Slglk2* overexpressing *SlGLK2* genotypes. Moreover, the interplay between *SlGLK2* and the auxins and cytokinin production and signalling was explored. The results expanded the knowledge regarding the complex regulatory network that controls chloroplast biogenesis and showed that SlGLK2 positively impacts tomato fruit quality in a light- and auxin-dependent manner.

## Material and methods

### Plant material, growth conditions and sampling

Experiments were carried out using *Solanum lycopersicum* cv. Micro-Tom, and depending on the experiment, different mutants in *SlGLK2* (wild-type allele) and *Slglk2* (mutant allele) backgrounds were used.

The PHY-deficient mutant *aurea* was chosen to explore the effect of PHY-mediated light perception on the regulation of *SlGLK2*. In *aurea*, a mutation on the PHYTOCHROMOBILIN SYNTHASE gene prevents the correct synthesis of the phytochromobilin, consequently leading to a global deficiency in functional PHYs [30, 31, 32]. To study the influence of auxin on the regulation of *SlGLK2*, *diageotropica* (*dgt*) was used, a mutant with reduced auxin sensitivity due to a mutation on *DIAGEOTROPICA* that encodes a cyclophilin [33]. The cytokinin effect on the regulation of *SlGLK2* was addressed in a transgenic plant overexpressing *Arabidopsis CYTOKININ OXIDASE 2* (*35S*::*CKX2*), resulting in low endogenous levels of cytokinins [34]. Moreover, transgenic plants expressing the reporter gene *uidA* (encoding for the β-GLUCURONIDASE enzyme, GUS) under control of the cytokinin (*ARR5*::*GUS*) and auxin (*DR5*::*GUS*) responsive promoters were used to evaluate the influence of SlGLK2 in hormonal activity.

Seeds from all tomato genotypes used were originally obtained from the germplasm collection maintained at ESALQ, Universidade de São Paulo (USP), Brazil (http://www.esalq.usp.br/tomato/).

All plants were greenhouse cultivated in 2L pots containing a 1:1 mixture of substrate (Plantmax HT, Eucatex, São Paulo, Brazil) and vermiculite supplemented with NPK (nitrogen phosphorus potassium) 10:10:10, dolomite limestone (MgCO_3_ + CaCO_3_) and magnesium thermophosphate (Yoorin Master, Yoorin Fertilizantes, Brazil), under controlled temperature (25 ± 3°C), daily automatically irrigation by capillarity, and natural light conditions (11.5 h/13 h photoperiod in winter/summer, respectively, and 250–350 μmolm^−2^ s^−1^ of incident photo-irradiance).

Fruits were collected at 12 h ± 1 h and sectioned in three parts; the most proximal and distal portions to the petiole were used, whereas the middle region was discarded. Only the pericarp (without placenta and locule walls) were used in the experiments. Fruits were harvested at six developmental stages: (i) immature green 3 (IG3, ~ 8 days post anthesis–dpa, actively growing fruits); (ii) immature green 5 (IG5, ~ 15 dpa, fruits at maximum size before ripening onset); (iii) mature green (MG, ~ 22 dpa, when the placenta displays a gelatinous aspect, transition to climacteric phase); (iv) breaker (Br, ~ 34 dpa, beginning of ripening, first signals of yellowish coloration); (v) orange (Br+3, three days after breaker stage, when the fruits display orange coloration); (vi) red ripe (Br+5, 5 days after breaker stage). A biological replicate was defined as a pool of fruits from at least five plants. All samples were frozen in liquid N_2_, powdered and stored at -80°C. For tocopherol content determination, samples were dried by lyophilization before extraction.

### qPCR analysis

RNA extraction, complementary DNA (cDNA) synthesis, primer design and qPCR assays were performed as described by [27]. Primer sequences used are detailed in S1 Table. qPCR reactions were carried out in a QuantiFlex Studio 6 real-time PCR system (Applied Biosystems) using 2X Power SYBR Green Master Mix reagent (Life Technologies) in a 14 μL final volume. Absolute fluorescence data were analysed using the LinRegPCR software package [35] in order to obtain the quantitation cycle (Cq) values and calculate PCR efficiency. Expression values were normalised against the geometric mean of two reference genes, *SlTIP41* and *SlEXPRESSED*, according to [27]. A permutation test lacking sample distribution assumption [36] was applied to detect statistical differences (P < 0.05) in expression ratios using the algorithms in the fgStatistics software package version 17/05/2012 [37].

### GUS activity fluorometric assay

Fruits from genotypes *DR5*::*GUS* and *ARR5*::*GUS* in *SlGLK2* and *Slglk2* background were powdered in liquid nitrogen and analysed through *in vitro* GUS activity quantitative assay, using methylumbelliferyl-β-D-glucuronide (MUG) according to [38] with the modifications described in [39].

### Chlorophyll and tocopherol quantification

Chlorophyll extraction was carried out as described in [40]. One mL of dimethylformamide (DMF) was added to 100 mg fresh weight of fruit samples. Then, samples were ice-cold sonicated for five min at 42 kHz and centrifugated at 9000 g for 10 min at room temperature and the supernatant collected. The procedure was repeated until total removal of green tissue colour. Spectrophotometer measurements were performed at 664 and 647 nm. Chlorophyll *a* content was estimated as (12*Abs 664)-(3,11*Abs 647), while chlorophyll *b* was calculated as (20,78* Abs 647)-(4,88* Abs 664); total chlorophyll was then obtained by adding the obtained values.

Tocopherols were extracted from approximately 25 mg dry weight as described in [25]. The samples were adjusted to 4 mL final volume. Aliquots of 3 mL were dried and dissolved in 200 μL of mobile phase composed of hexane/tert-butyl methyl ether (90:10). Chromatography was carried out on a Hewlett-Packard series 1100 HPLC system coupled with a fluorescence detector (Agilent Technologies series 1200) on a normal-phase column (LiChrosphere 100 Diol Si; 250 mm x 4.0 mm, 5 μm; Agilent Technologies, Germany) at room temperature with the mobile phase running isocratically at 1 mL min-1. α-, β-, γ- and δ-tocopherol were detected by excitation at 295 nm, and fluorescence was quantified at 330 nm.

### Transgenic plant generation

*SlGLK2* full-length cDNA was amplified (primers described in S1 Table), cloned into pK7WG2D,1 [41] and introduced in *Solanum lycopersicum* (cv Micro Tom) *aurea Slglk2* (*au-Slglk2*) mutant background following the protocol described in [42]. *SlGLK2*-overexpressing transgenic lines (L2, L7 and L8) were confirmed by genomic PCR and qPCR (primers described in S1 Table). The experiments were performed with T1 plants.

### Total protein quantification and Western Blot

Approximately 400 mg of immature green fruits were frozen in liquid nitrogen, powdered and lyophilised. The homogenized tissue was resuspended in extraction buffer (1:1 w/v ratio) containing 10 mM KCl, 5 mM MgCl 2, 400 mM sucrose, 10 mM β-mercaptoethanol, 100 mM Tris-HCl, pH 8, 10% glycerol (v/v), 1 mM PMSF [43] and 1:100 Protease Inhibitor Cocktail (Sigma-Aldrich # P9599) and centrifuged twice at 12,000 RPM for 15 min at 4°C. The supernatant was collected and quantified using the Bradford assay [44]. Approximately 80 μg of total protein extracts were separated by SDS/PAGE on a 12% (w/v) acrylamide (30% acrylamide/Bis Solution, 29:1; Bio-Rad) gel and transferred on a 0.45-mm nitrocellulose membrane (Bio-Rad # 1620115). The membrane was stained with Ponceau red to assess equal transfer. Blotted membrane was blocked in Tris-buffered saline containing 0.1% (v/v) Tween 20 (TBS-T) with 5% (w/v) dry milk (Biorad # 1706406) for 1 h and 30 min at 37°C, then washed three times for 5 min with TBS-T and incubated with a specific polyclonal antibody raised against synthetic peptide based on specific amino acid sequence of SlGLK2 (peptide sequence: CSLSYKNERENYD) (FastBio, Brazil). After incubation with the primary antibody, the membrane was washed and subsequently incubated with Alkaline phosphatase-coupled anti-rabbit secondary antibody (Sigma-Aldrich # A-3687) for 3 h at room temperature. AP Conjugate Substrate Kit (Bio-Rad # 1706432) was used for detection.

### Transmission electron microscopy and chloroplast counting

For plastid ultrastructure analysis, samples of immature green fruits at IG5 stage were fixed in glutaraldehyde 2.5% (v/v) in 100 mM sodium phosphate buffer (pH 7.2) for 2 hours, post-fixed in sodium tetroxide 2% (w/v) in the same buffer for 2 hours and treated with tannic acid 1% (w/v) in sodium phosphate buffer 50 mM (pH 7.2) for 16 hours. Dehydration was performed gradually in acetone, and finally, samples were embedded in Spurr resin (Electron Microscopy Science #14300). Ultra-thin sections were stained with uranyl acetate [45] and citrate [46] and visualized under transmission electron microscopy JEOL model JEM1011. The size of the chloroplasts was measured from the transmission electron microscopy images.

To count chloroplasts, slides from immature green fruits at IG5 stage were prepared as described by [47] and analysed in optical Axio Imager M2 microscope. Chloroplasts per cell were manually counted.

### Soluble sugars and total soluble solids quantification

For quantification of soluble sugars, 200 mg fresh weight of powdered fruit pericarp was extracted with 1 mL ethanol 80% (v/v) four times as described by [48]. Approximately 1 mL of the extract was vacuum dried in a SpeedVac system and resuspended in 1 mL of ultra-pure water. Glucose, fructose and sucrose were quantified by high-performance anion exchange chromatography with pulsed amperometric detection (HPAEC-PAD; Dionex, Sunnyvale, CA, USA) using a Carbopac PA1 column (250 x 4 mm, 5 μm particle size, Dionex) in an isocratic run with 18 mM NaOH as mobile phase. Content of each sugar was calculated using standard curves made with pure glucose, fructose and sucrose. Total soluble solids were measured in ripe fruits with a refractometer DR201-95 (Kruss).

### Data analysis

Differences in parameters were statistically evaluated using Infostat software v. 2016 [37]. When the data set showed homoscedasticity, ANOVA analysis was performed to compare values along development in the same genotype (*P*<0.05). In the absence of homoscedasticity, non-parametric analysis was performed using the Kruskal Wallis test (*P*<0.05).

## Results

### The deficiency in PHY-mediated light perception alters the temporal, but not the spatial, pattern of *SlGLK2* mRNA accumulation in fruits

**Fig 1:**
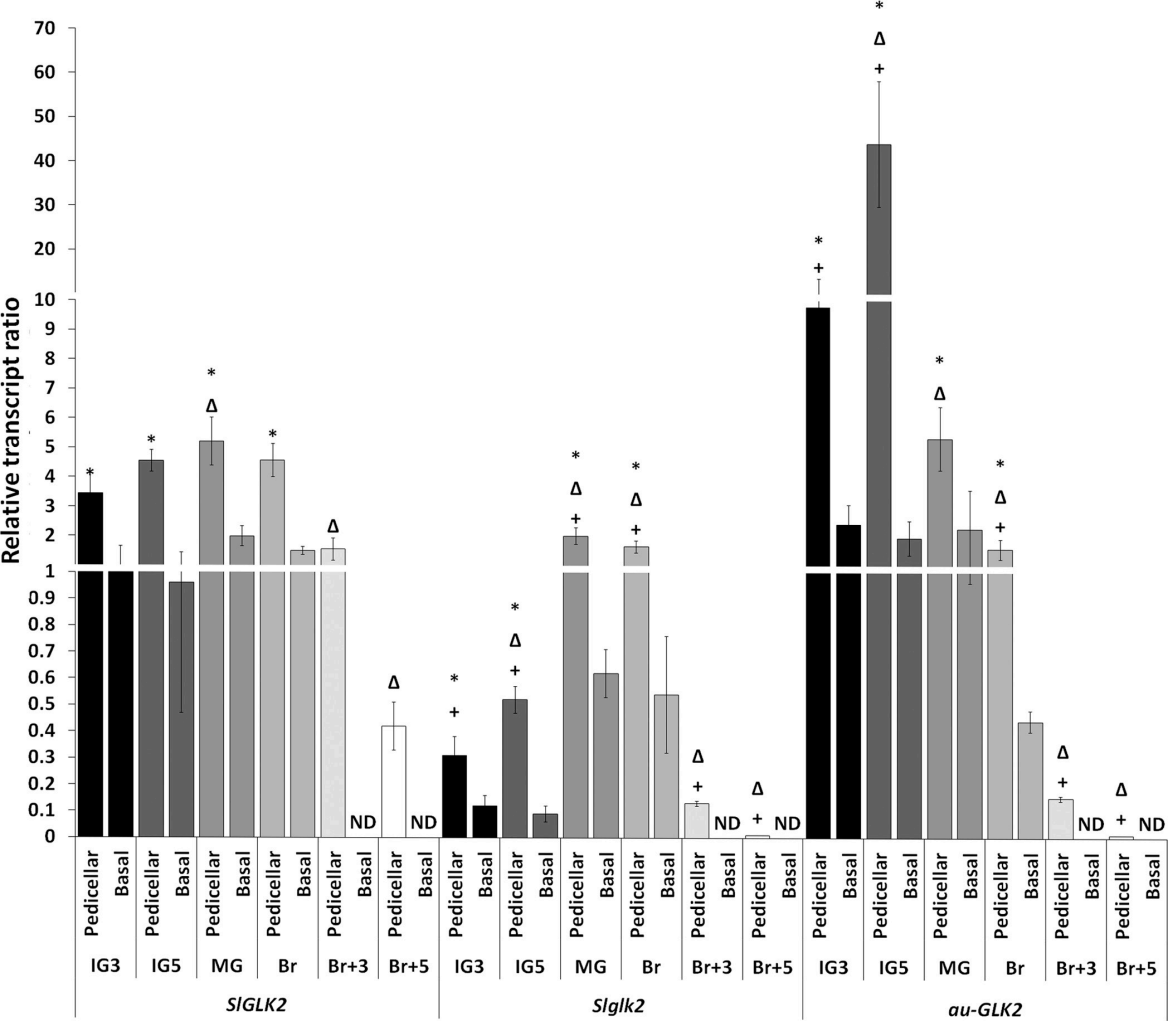
Spatial and temporal pattern of *SlGLK2* transcript abundance. Histogram representation of *SlGLK2* relative mRNA levels in wild-type (*SlGLK2*), *Slglk2* and *au-SlGLK2* mutant genotypes. Values represent means ± SE of at least three biological replicates normalised against the basal IG3 sample from *SlGLK2* genotype. Statistically significant differences between the pedicellar and basal samples are indicated by asterisks (*P* < 0.05). Within the pedicellar samples from each genotype, statistically significant differences to the respective IG3 sample are indicated by triangles (*P* < 0.05). Within the pedicellar samples of each stage, statistically significant differences to *SlGLK2* genotype are indicated by plus signal (*P* < 0.05). ND: Not detected. The entire set of data is presented in S2 Table.

To address whether the presence of the mutant allele, *Slglk2*, or the global deficiency in functional PHYs disrupt the spatial and temporal *SlGLK2* expression pattern, three genotypes were analysed, the wild-type harbouring *SlGLK2* allele (*SlGLK2*), *Slglk2* mutant (*Slglk2*), and PHY-deficient *aurea* (*au)* mutant harbouring the wild-type *SlGLK2* allele (*au-SlGLK2*). The mRNA abundance of *SlGLK2* was addressed at the pedicellar and the bottom part of the fruit at immature green (IG3 and IG5), mature green (MG), breaker (Br) and three and five days after breaker (Br+3 and Br+5, respectively) stages. For all genotypes, *SlGLK2* transcripts were found in both portions until the onset of ripening (Br), although at least 2.5-fold more abundantly in the pedicellar portion of the fruits, becoming undetectable in the bottom part at Br+3 and Br+5 stages (Fig 1, S2 Table). Thus, the expression gradient along fruit longitudinal axis was maintained in the absence of a functional SlGLK2 protein (in *Slglk2* mutant) or when PHY-mediated light perception was impaired (in *au*-*SlGLK2* mutant). Regarding temporal expression profile in the pedicellar portion of the fruit, in *SlGLK2* wild-type and *Slglk2* mutant genotypes the mRNA amount peaked at the MG stage declining gradually as ripening progressed. However, a different profile was observed in *au-SlGLK2* genotype fruits, in which the highest amount of *SlGLK2* transcripts was verified at IG5 stage (Fig 1, S2 Table). When comparing the pedicellar portion of the distinct genotypes, the mRNA of *Slglk2* mutant allele accumulated at levels lower than those of *SlGLK2* wild-type allele, throughout fruit development and ripening. The deficiency in the PHY-mediated light perception characteristic of the *au-SlGLK2* led to increased levels of *SlGLK2* transcript at immature green stages and decreased amounts from Br stage onwards compared to the wild-type genotype (Fig 1, S2 Table). Thus, in all genotypes, *SlGLK2* mRNA peaks during initial fruit development and declines towards ripe stages. These results indicate that, while the spatial gradient along the fruit longitudinal axis is maintained, the temporal expression pattern of *SlGLK2 locus* along fruit development and ripening is affected by PHY-mediated signalling.

### *SlGLK2* is negatively regulated by auxin and enhances cytokinin responsiveness

Aiming to improve the knowledge about the SlGLK2 signalling network, the interplay between this transcription factor and the auxins and cytokinins production and signalling was investigated.

**Fig 2:**
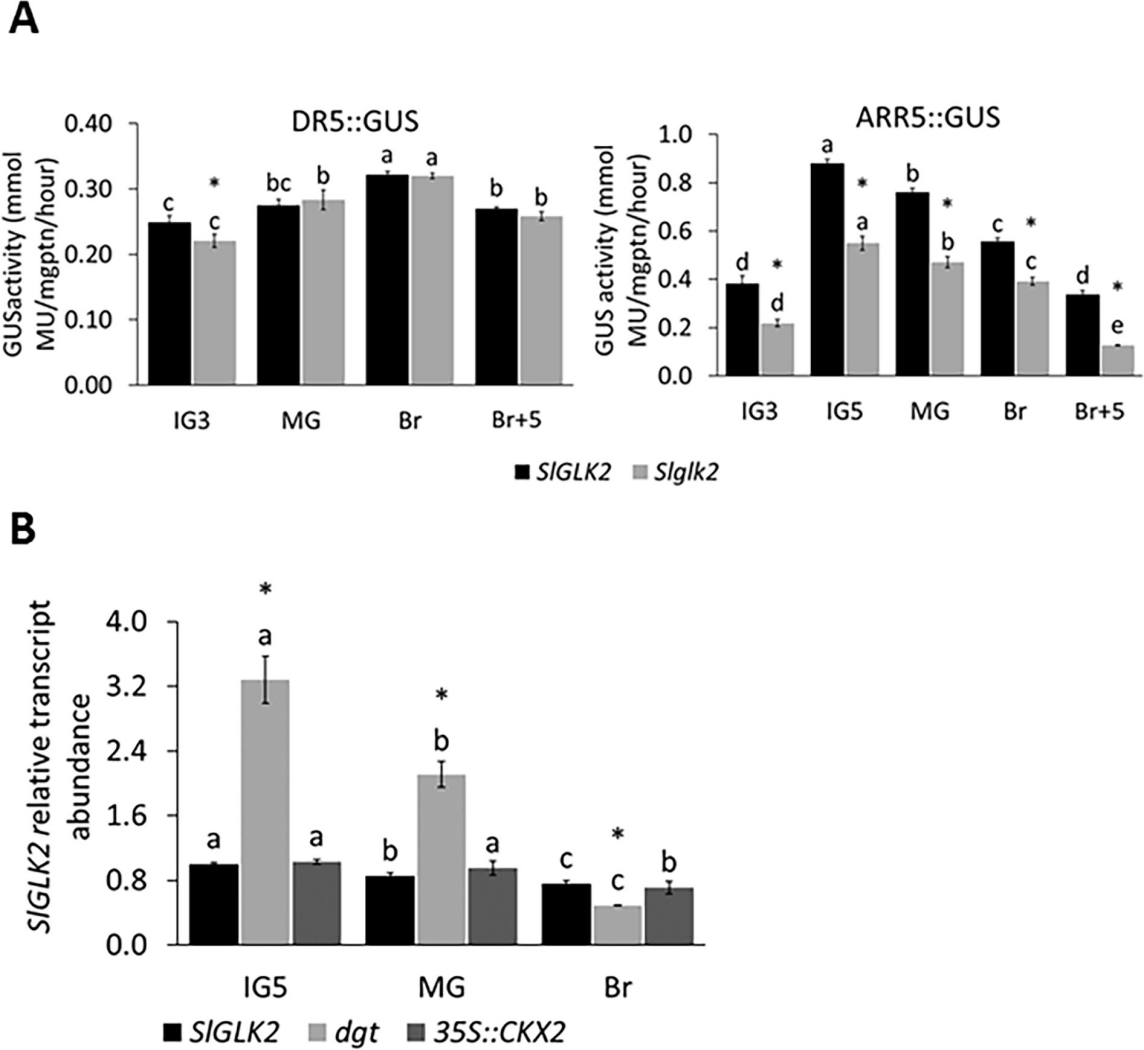
Interplay between *SlGLK2* and auxin and cytokinin production and signalling. (A) Auxin and cytokinin responsiveness in genotypes encoding functional (*SlGLK2*) and truncated (*Slglk2*) SlGLK2 proteins. Responsiveness was addressed by evaluating the activity of the GUS reporter, whose expression was controlled by the auxin- or cytokinin-responsive promoter *DR5* and *ARR5*, respectively. Different letters indicate statistically significant differences between stages within each genotype (*P* < 0.05). Asterisks denote statistically significant differences between genotypes within the same fruit stage (*P* < 0.05). Values represent mean ± SE of at least three biological replicates. (B) Relative transcript value of *SlGLK2* along fruit development and ripening in wild-type (*SlGLK2)*, auxin insensitive (*dgt*) and low-cytokinin (*35S*::*CKX2)* genotypes. Different letters indicate statistically significant differences between stages within each genotype (*P* < 0.05). Asterisks denote statistically significant differences between genotypes within the same fruit stage in comparison to the *SlGLK2* sample (*P* < 0.05). Values represent mean ± SE of at least three biological replicates.

The eventual impact of *SlGLK2* wild-type allele on hormonal activity was evaluated by analysing the activity of GUS reporter enzyme expressed under the control of the *DR5* and *ARR5* promoters, which are responsive to auxins and cytokinins, respectively. This fluorometric assay uses 4-methylumbeliferyl-β-D-glucuronide (MUG) as substrate, which is hydrolysed by GUS in glucuronic acid and the fluorescent 4-methylumbeliferone, providing a precise quantitative data of GUS activity [38]. While no marked differences were found in GUS activity between *DR5*::*GUS-SlGLK2* and *DR5*::*GUS-Slglk2*, GUS activity was higher in the fruits from *ARR5*::*GUS-SlGLK2* than in those from *ARR5*::*GUS-Slglk2* in all stages analysed (Fig 2A). These data indicate that SlGLK2 has a positive influence on cytokinin signalling, evidenced by the increment in *ARR5* promoter activity in the presence of the wild-type allele.

Subsequently, the influence of both hormones on the transcriptional regulation of SlGLK2 was investigated by analysing the *SlGLK2* transcript abundance in the auxin-resistant *diageotropica* (*dgt*) mutant and the cytokinin-deficient *35S*:*CKX2 (CYTOKININ OXIDASE/DEHYDROGENASE 2)* genotype (Fig 2B). The reduction in cytokinin content of the *35S*:*CKX2* genotype did not affect the mRNA abundance of *SlGLK2*. However, the reduced auxin responsiveness characteristic of the *dgt* mutant significantly promoted *SlGLK2* transcript accumulation in immature and mature green stages. Thus, while the presence of *SlGLK2* wild-type allele increases the fruit tissue responsiveness to cytokinins, the expression of *SlGLK2* is negatively regulated by auxins.

### Tocopherol metabolism is affected by SlGLK2 and PHY-mediated light signalling

**Fig 3:**
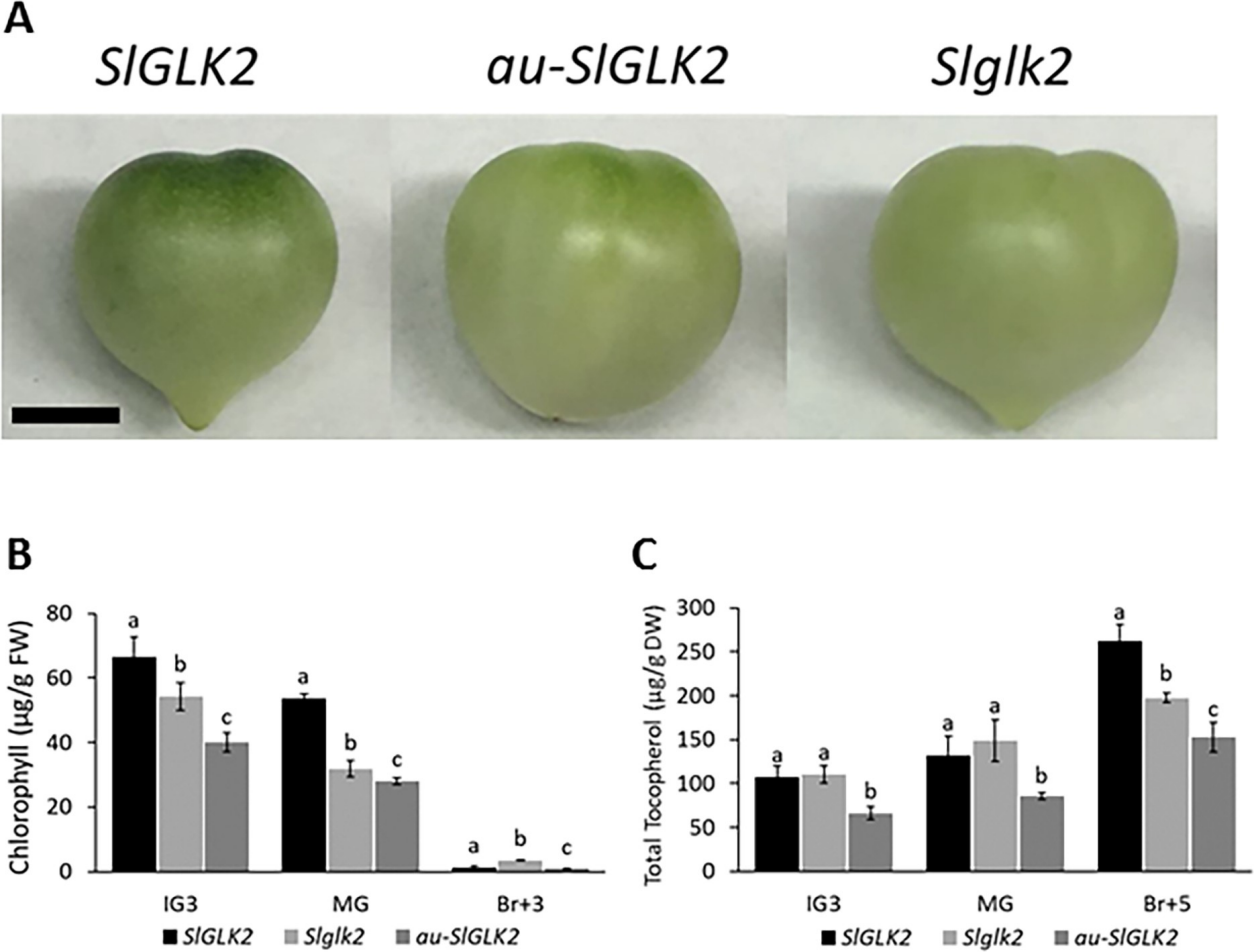
Phenotype, chlorophyll and tocopherol content of fruits. Phenotype of fruits at IG5 stage from *SlGLK2*, *au-SlGLK2* and *Slglk2* genotypes. Scale bar = 1 cm **(A)**. Chlorophyll **(B)** and total tocopherol **(C)** content in the pedicellar portion of fruits of wild-type (*SlGLK2*), *Slglk2* and *au-SlGLK2* mutant genotypes. Different letters indicate statistically significant values between genotypes within each stage (*P* < 0.05). Values represent mean ± SE of at least three biological replicates.

Since tocopherol is synthesized in the plastids and its production has been robustly demonstrated to be linked to chlorophyll metabolism in tomato fruits [25, 26, 27], the effect of *SlGLK2*, and its PHY-dependent transcriptional regulation demonstrated above, on tocopherol content was investigated. As expected, *SlGLK2* genotype displayed higher levels of chlorophyll than *Slglk2* and *au-SlGLK2* genotypes in green stages of fruit development (Fig 3A). Interestingly, the presence of *SlGLK2* wild-type allele in *aurea* background was not enough to recover chlorophyll content, which did not surpass those detected in the *Slglk2* mutant genotype (Fig 3B). Regarding total tocopherol, green fruits, *i*.*e*. IG3 and MG, of *SlGLK2* and *Slglk2* had similar levels, which were higher than *au-SlGLK2*. In ripe fruits, *i*.*e*. Br+5, when chlorophyll was degraded, all genotypes showed increased total tocopherol content than the respective green stages. This profile correlated with chlorophyll contents at green stages, where *SlGLK2* wild-type genotype and the *au-SlGLK2* mutant accumulated the highest and the lowest total tocopherol content, respectively (Fig 3C). When tocopherol forms were individually analysed (S3 Table), it became evident that all detectable tocopherol forms in green stages, except for γ-tocopherol in IG3 stage, of *au-SlGLK2* fruits were less abundant when compared to *SlGLK2* wild-type fruits. This trend was particularly conspicuous for α-tocopherol, which is the most abundant form in tomato. At the ripe stage, fruits from *au-SlGLK2* exhibited a reduction in all tocopherol forms, while, *Slglk2* showed decreased levels of γ- and δ-tocopherol in comparison to *SlGLK2* wild-type genotype. It is worth mentioning that the β, δ and γ forms little contribute to the total amount and vary between organs and tomato varieties. The minor tocopherol forms are barely detected or remain below the HPLC detection level (as the case of δ in MG and IG3) [25, 27].

**Fig 4:**
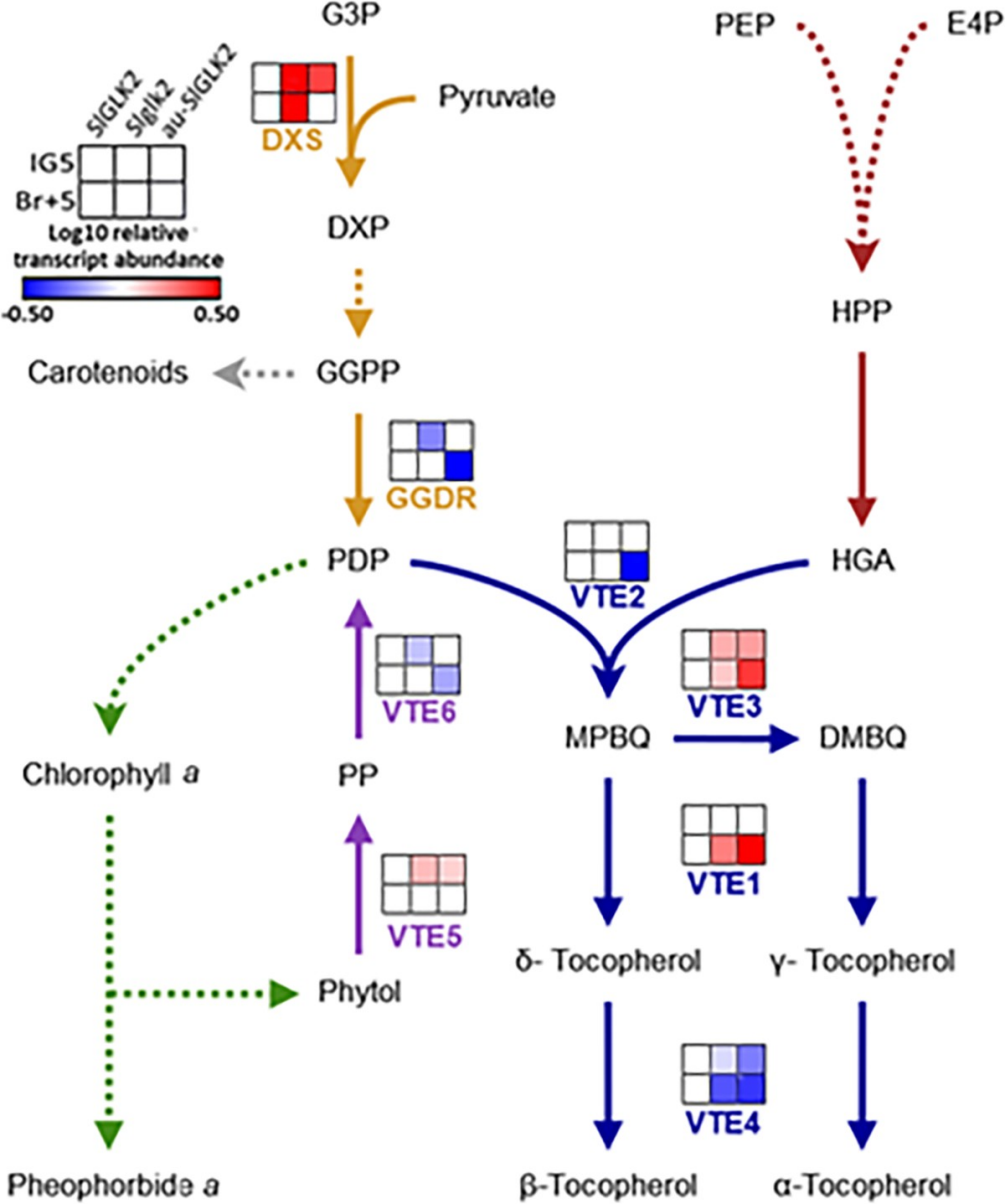
Transcript profile of tocopherol-related encoding genes. Heatmap representation of the mRNA abundance of tocopherol-related encoding genes in the pedicellar portion of immature green (IG5) and ripe (Br+5) fruits of wild-type (*SlGLK2*), truncated SlGLK2-encoding (*Slglk2*) and *au-SlGLK2* mutant genotypes. Coloured squares represent statistically significant differences in relation to the respective wild-type (*SlGLK2*) sample (*P* < 0.05). Values represent mean of at least three biological replicates. Relative transcript values are detailed in S4 Table. Methylerythritol phosphate pathway (orange), shikimate pathway (red), chlorophyll metabolism (green), phytol recycling (purple) and tocopherol biosynthesis (blue). Dotted lines indicate that intermediate steps were omitted. Enzymes: *DXS*: *1-DEOXY-D-XYLULOSE-5-P SYNTHASE; GGDR*: *GERANYLGERANYL DIPHOSPHATE REDUCTASE; VTE1*: *TOCOPHEROL CYCLASE; VTE2*: *HOMOGENTISATE PHYTYL TRANSFERASE; VTE3*: *2*,*3-DIMETHYL-5-PHYTYLQUINOL METHYL TRANSFERASE; VTE4*: *TOCOPHEROL γ-METHYL TRANSFERASE; VTE5*: *PHYTOL KINASE; VTE6*: *PHYTYL-PHOSPHATE KINASE*. Metabolites: G3P: glyceraldehyde 3-phosphate; DXP: 1-deoxy-D-xylulose-5P; GGDP: geranylgeranyl-2P; PDP: phytyl diphosphate; HGA: homogentisate; HPP: hydroxyphenylpyruvate; PEP: phosphoenolpyruvate; E4P: erythrose 4-phosphate; MPBQ: 2-methyl-6-phytylquinol; DMBQ: 2,3-dimethyl-5-phytylquinol; PP: phytyl phosphate.

To better understand the alterations in chlorophyll and tocopherol content described above, the mRNA of genes that are known to be transcriptionally regulated and contribute in determining ripe fruit tocopherol content [25, 26] was profiled (Fig 4, S4 Table). The transcriptional profile included *SlDXS* and *SlGGDR* from the methylerythritol-phosphate pathway, *SlVTE1*, *SlVTE2*, *SlVTE3* and *SlVTE4* from tocopherol biosynthesis, and *SlVTE5* and *SlVTE6* involved in phytol recycling. At IG5 stage, just before the onset of ripening, the reduction of the mRNA levels of *SlDXS* and *SlGGDR* in the absence of functional SlGLK2 limits the phytol availability, explaining the decreased chlorophyll content in *Slglk2* genotype. In *au-SlGLK2* mutant the impairment in chlorophyll tetrapyrrolic ring biosynthesis and in PHY-mediated signalling that attenuates chloroplast differentiation [49] explain the reduction in chlorophyll and tocopherol level in green fruits. Therefore, the altered expression pattern of *SlVTE3*, *SlVTE4* and *SlVTE5* might be a compensatory effect in response to the reduction in tocopherol levels.

As ripening proceeded, at Br+5 stage the fruits from *Slglk2* genotype displayed no alteration in the transcript levels of *SlGGDR* nor in phytol recycling-related genes; therefore, the reduced content of chlorophyll in the green fruits might have decreased the pool of free phytol during ripening, explaining the reduced levels of tocopherol. Again, *SlVTE1* and *SlVTE3* upregulation and *SlVTE4* downregulation might be a compensatory response to the reduced phytol availability in *Slglk2* genotype. In the case of *au-SlGLK2*, besides the changes observed in *Slglk2*, reduced levels of *SlVTE2*, *SlVTE6*, *SlDXS* and *SlGGDR* mRNA were verified, contributing to the decrease in tocopherol levels together with the reduced chlorophyll content.

Collectively, these results suggest that both SlGLK2 and PHY-mediated light perception affect the tocopherol biosynthesis and accumulation in tomato fruits, not only through their impact on chlorophyll biosynthesis and, consequently, on the availability of chlorophyll-derived phytol, but also by the transcriptional regulation of enzyme-encoding genes involved in tocopherol biosynthetic, phytol recycling and methylerythritol-phosphate pathways.

### *SlGLK2* overexpression in PHY-deficient plants promotes fruit chlorophyll accumulation and chloroplast biogenesis in green fruits and restores wild-type levels of tocopherol in ripe fruits

**Fig 5:**
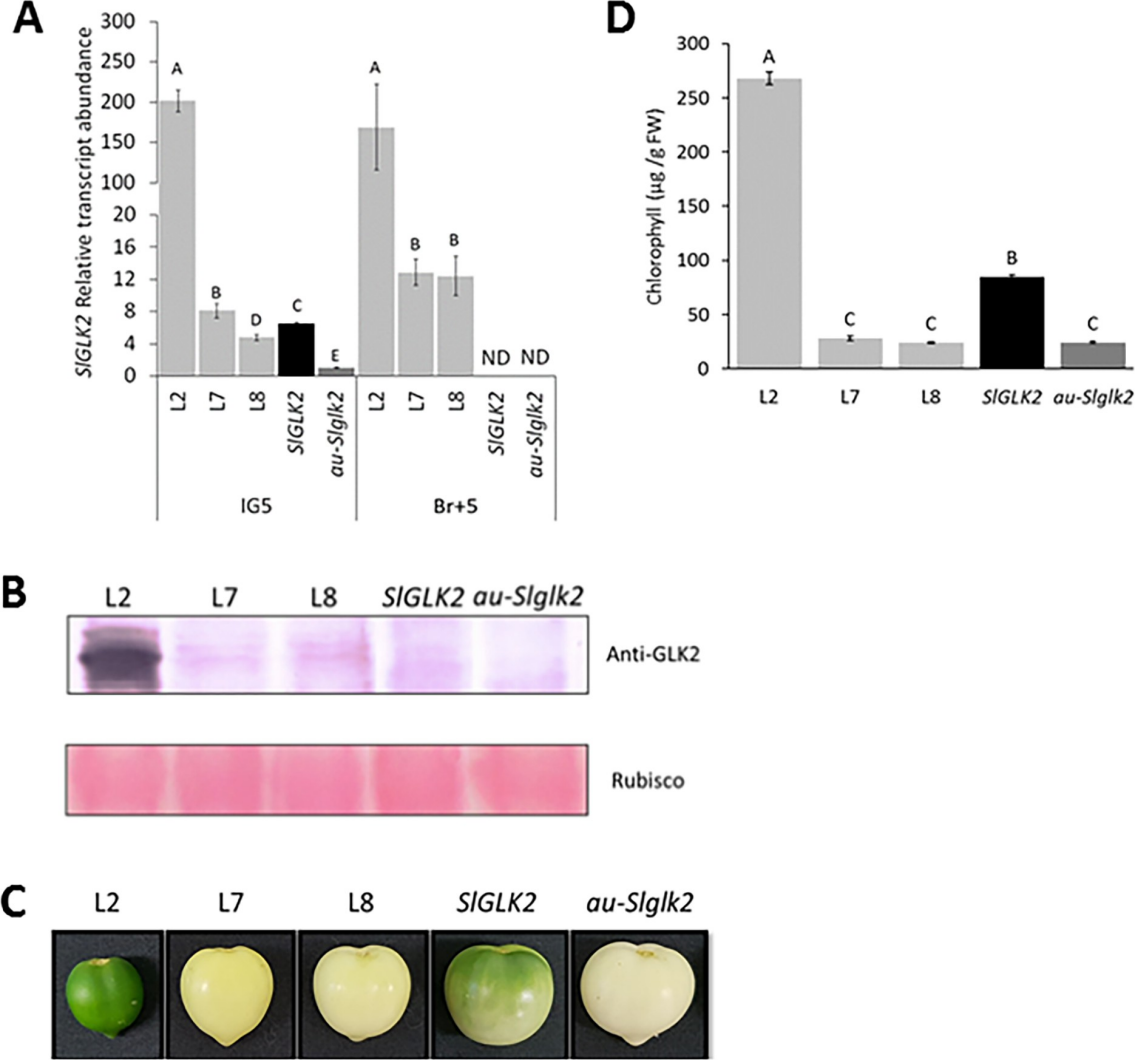
Characterisation of *SlGLK2* overexpressing lines in *au-Slglk2* background. (A) *SlGLK2* relative transcript ratio in the pedicellar region of IG5 fruits from control genotypes (*SlGLK2* and *au-Slglk2)* and *SlGLK2-*overexpressing lines (L2, L7 and L8). Letters indicate statistically significant differences among the genotypes (*P* < 0.05). Values are normalised against *au-Slglk2* sample and represent mean ± SE of at least three biological replicates. (B) Detection of SlGLK2 protein (34.4 KDa) by Western blot in the pedicellar portion of IG5 fruits from control genotypes (*SlGLK2* and *au-Slglk2)* and *SlGLK2-*overexpressing lines (L2, L7 and L8). (C) Fruit phenotypes from control genotypes (*SlGLK2* and *au-Slglk2)* and *SlGLK2-*overexpressing lines (L2, L7 and L8). (D) Chlorophyll content in the pedicellar portion of IG5 fruits from control genotypes (*SlGLK2* and *au-Slglk2)* and *SlGLK2-*overexpressing lines (L2, L7 and L8). Letters indicate statistically significant differences among the genotypes (*P* < 0.05). Values represent mean ± SE of at least three biological replicates.

Chlorophyll levels in green fruits before the onset of ripening directly affects tocopherol content in ripe fruits (Fig 3B and 3C) [25]. Thus, to evaluate whether *SlGLK2* could promote chloroplast differentiation and tocopherol accumulation despite the impairment in PHY-mediated light perception, transgenic lines constitutively overexpressing *SlGLK2* wild-type allele in *aurea Slglk2* (*au-Slglk2*) genetic background were generated. Three transgenic lines, namely L2, L7 and L8, which displayed at least 4-fold increases in *SlGLK2* transcript abundance in IG5 fruits (Fig 5A), were selected for further characterisation. The amount of SlGLK2 protein was estimated at IG5 stage and, while undetected in the untransformed *au-Slglk2* that harbours the mutated allele encoding a truncated protein, it was only faintly detected in L7, L8 and *SlGLK2* wild-type fruits. However, the SlGLK2 protein was conspicuously detected in L2, whose mRNA levels surpassed those found in the untransformed *au-Slglk2* and *SlGLK2* genotypes approximately 200- and 28-fold, respectively (Fig 5B). Phenotypically, both L7 and L8 immature fruits maintained the chlorotic aspect characteristic of the *aurea* mutation, while L2 developed fruits with uniform dark green colour (Fig 5C). It is worth mentioning that this phenotype was not exclusively of L2, a fourth transgenic line, namely L6, also displayed dark green fruits. However the T0 plant produced parthenocarpic fruits exhibiting only aborted seeds, thus, we were unable to further analyse this line (S1 Fig).

In line with the fruit colour phenotypes observed, the chlorophyll content in IG5 fruits of the L2 transgenic line was markedly higher than in untransformed *au-Slglk2* genotype, even when compared to *SlGLK2* wild-type genotype (Fig 5D). No differences were found in chlorophyll content between the L7 and L8 transgenic lines and the untransformed genotype. The distinct phenotype of L2, that was also displayed by L6 and is in agreement to what was reported to wild-type tomato fruits overexpressing *AtGLK2* [12], together with the expression and the western blot results allowed us to conclude that the only transgenic line that indeed accumulates higher levels of the SlGLK2 protein is L2; therefore, only this line was further characterised.

**Fig 6:**
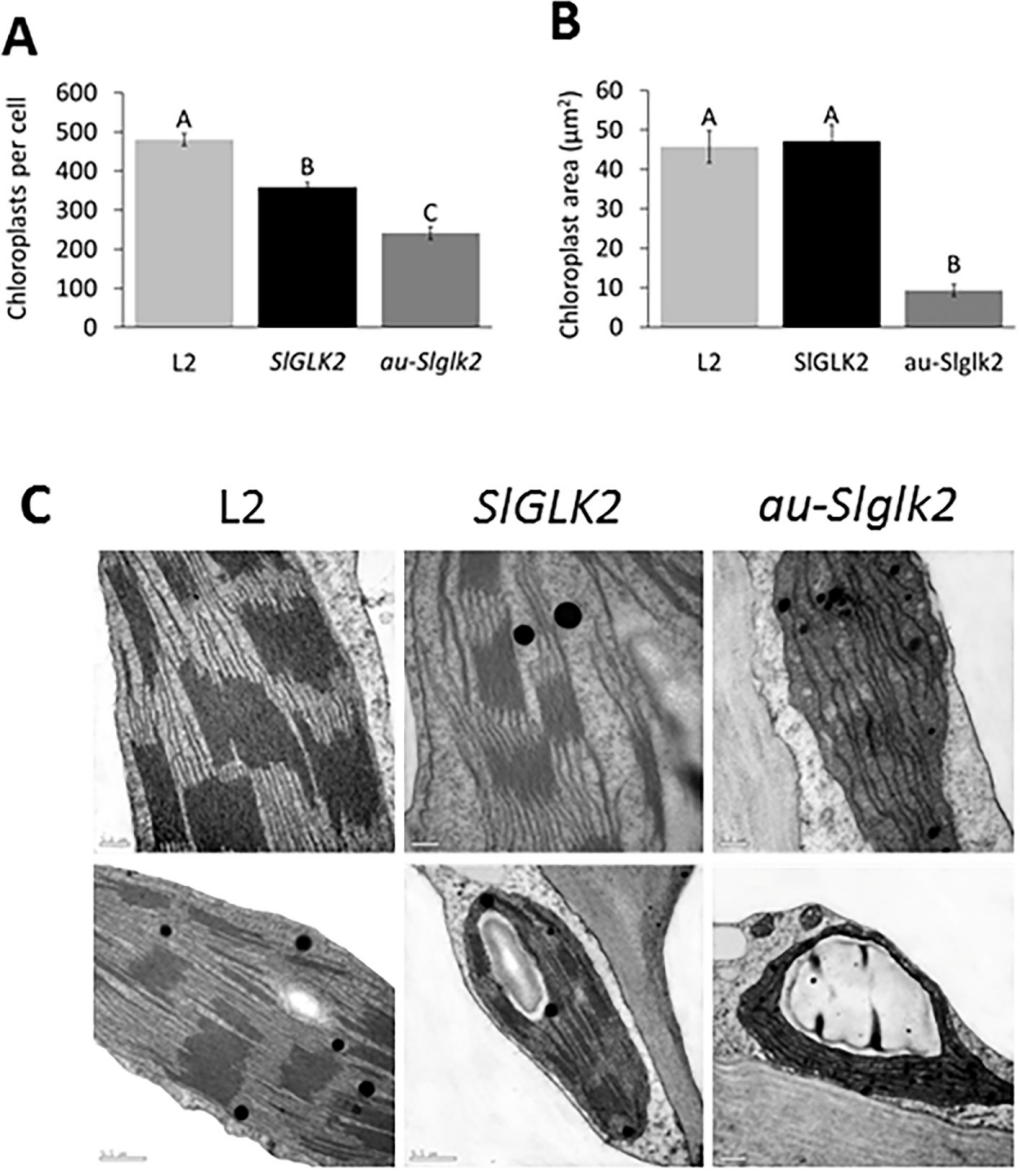
Chloroplast number and structure in *SlGLK2* overexpressing lines. **(A)** and **(B)** Number and area of chloroplasts at the pedicellar portion of IG5 fruits of untransformed genotypes (*SlGLK2* and *au-Slglk2)* and transformed *au-Slglk2 SlGLK2-*overexpressing L2 line. Letters indicate statistically significant differences among the genotypes (*P* < 0.05). Values represent mean ± SE of at least 10 biological replicates. **(C)** Chloroplast ultrastructure of IG5 fruits of untransformed genotypes (*SlGLK2* and *au-Slglk2)* and transformed *au-Slglk2 SlGLK2-*overexpressing L2 line. Scale bar = 0.5 μM (above) or 0.2 μM (below).

To address whether the increment in chlorophyll content might reflect an alteration in chloroplast differentiation, chloroplast number and ultrastructure were evaluated in IG5 fruits. Immature fruits from L2 transgenic line had more chloroplasts than both the untransformed *au-Slglk2* and *SlGLK2* genotypes (Fig 6A). Ultrastructural analysis revealed *SlGLK2* overexpression in *aurea-Slglk2* background was able to restore chloroplast size to that observed in the wild type *SlGLK2* genotype (Fig 6B). Moreover, fruit chloroplasts from L2 line exhibited grana with more abundantly stacked thylakoids and notably fewer starch grains compared to *au-Slglk2* and *SlGLK2* genotypes (Fig 6C).

**Fig 7:**
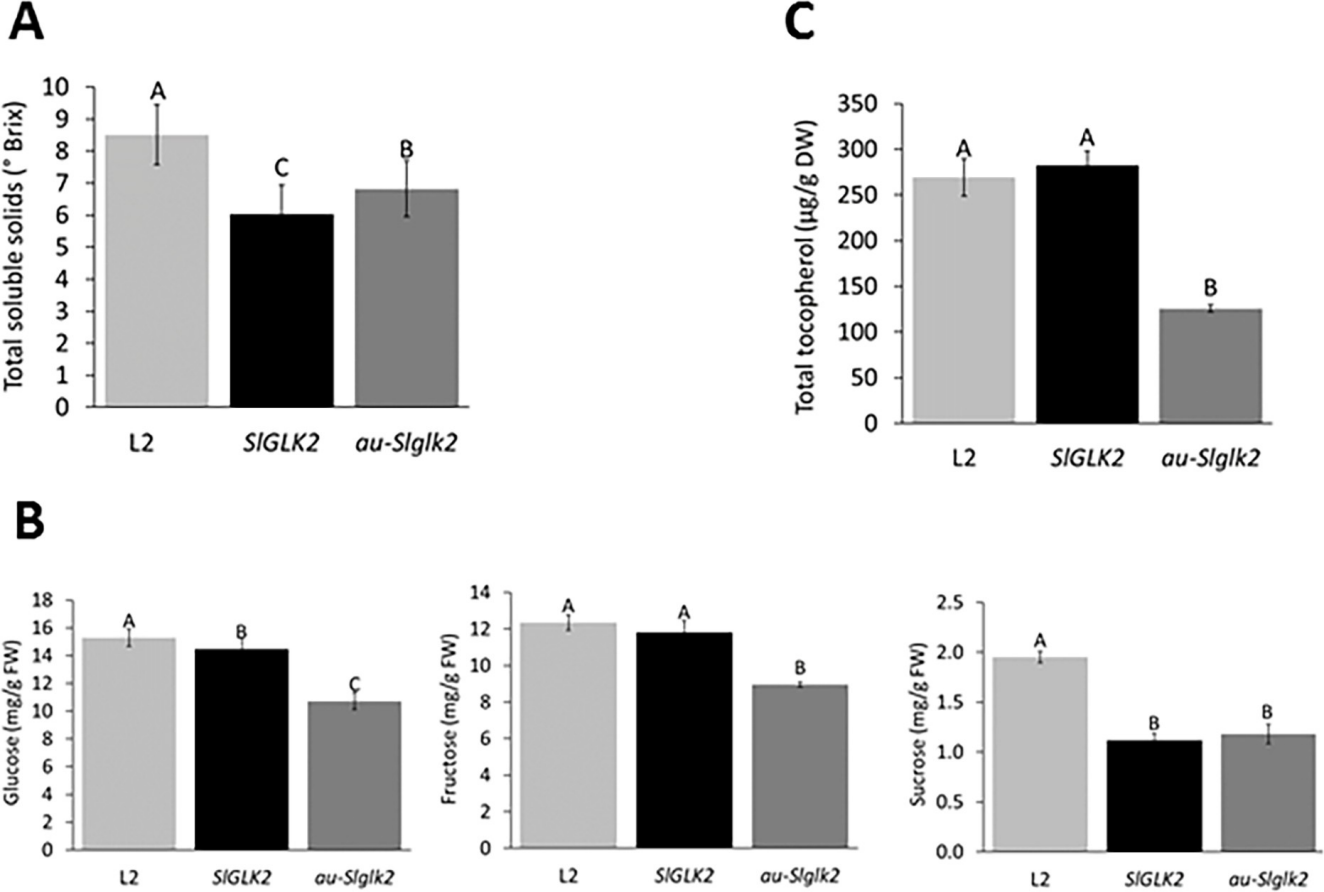
Sugar and tocopherol content in ripe fruits from *SlGLK2* overexpressing line. (A) Total soluble solids (°BRIX) in Br+5 ripe fruits from untransformed genotypes (*SlGLK2* and *au-Slglk2)* and *SlGLK2-*overexpressing L2 line. Letters indicate statistically significant differences among the genotypes (*P* < 0.05). Values represent mean ± SE of at least 12 biological replicates. Soluble sugars (glucose, fructose and sucrose) (B) and tocopherol content (C) in the pedicellar portion in Br+5 ripe fruits from untransformed genotypes (*SlGLK2* and *au-Slglk2)* and *SlGLK2-*overexpressing L2 line. Letters indicate statistically significant differences among the genotypes (*P* < 0.05). Values represent mean ± SE of at least three biological replicates.

To investigate whether the alterations in chloroplast number and internal structure in immature fruits from L2 overexpressing line would restore the quality of the edible fruits, we measured plastid metabolic activity products in Br+5 stage. L2 ripe fruits exhibited great amounts of total soluble solids than those observed in both control genotypes (Fig 7A), which correlates with the increment in sucrose also detected in the overexpressing line (Fig 7B). Additionally, the tocopherol content in ripe fruits from the L2 genotype increased up to the value observed in *SlGLK2* genotype (Fig 7C; S5 Table). Tocopherol profiling in ripe fruits revealed that *SlGLK2* overexpression led greater δ- and γ-tocopherol accumulation compared to both *au-Slglk2* and *SlGLK2* control genotypes.

**Fig 8:**
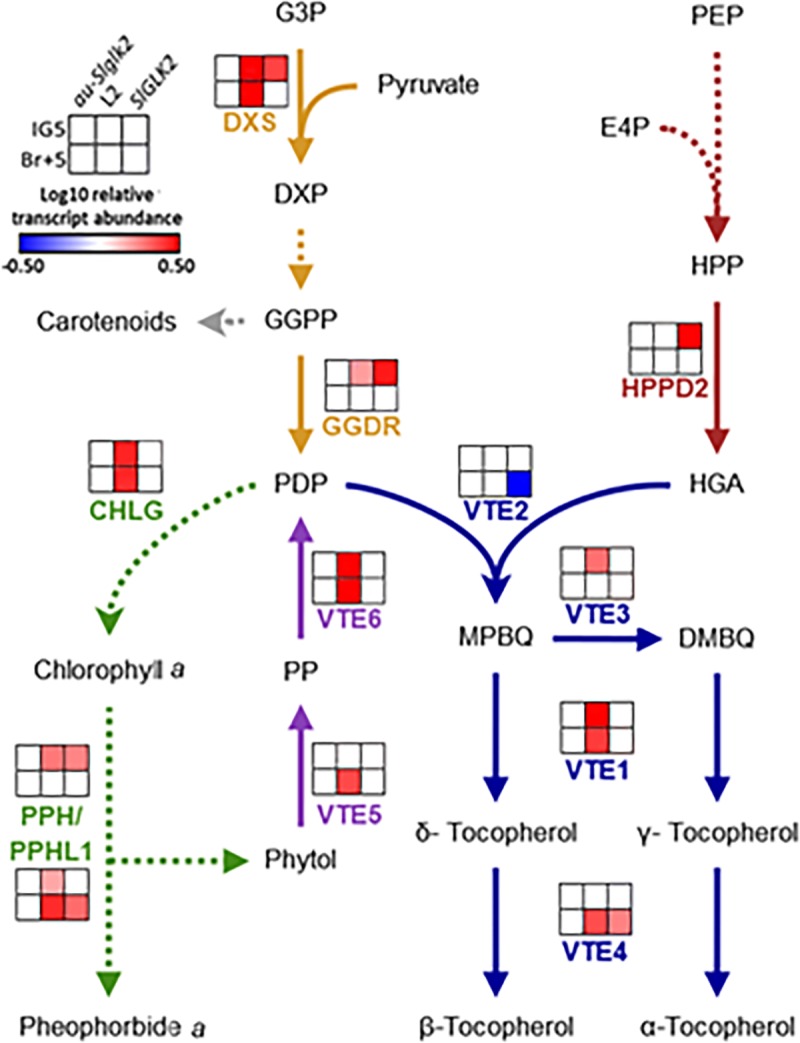
Transcript profile of tocopherol-related encoding genes in fruits from *SlGLK2* overexpressing line. Heatmap representation of the mRNA abundance of tocopherol-related encoding genes in the pedicellar portion of immature green (IG5) and ripe (Br+5) fruits of untransformed genotypes (*SlGLK2* and *au-Slglk2)* and transformed *au-Slglk2 SlGLK2-*overexpressing L2 line. Values are normalised against the respective *au-Slglk2* sample. Coloured squares indicate statistically significant differences compared to *au-Slglk2* (*P* < 0.05). Values represent mean of at least three biological replicates. Relative transcript values are described in S6 Table. Methylerythritol phosphate pathway (orange), shikimate pathway (red), chlorophyll metabolism (green), phytol recycling (purple) and tocopherol biosynthesis (blue). Dotted lines indicate that intermediate steps were omitted. Enzymes: *DXS*: *1-DEOXY-D-XYLULOSE-5-P SYNTHASE; HPPD2*: *4-HYDROXYPHENYLPYRUVATE DIOXYGENASE (2); GGDR*: *GERANYLGERANYL DIPHOSPHATE REDUCTASE; VTE1*: *TOCOPHEROL CYCLASE; VTE2*: *HOMOGENTISATE PHYTYL TRANSFERASE; VTE3*: *2*,*3-DIMETHYL-5-PHYTYLQUINOL METHYL TRANSFERASE; VTE4*: *TOCOPHEROL Γ -METHYL TRANSFERASE; VTE5*: *PHYTOL KINASE; VTE6*: *PHYTYL-PHOSPHATE KINASE; PPH*: *PHEOPHYTINASE; PPHL1*: *PHEOPHYTINASE LIKE-1; CHLG*: *CHLOROPHYLL SYNTHASE*. Metabolites: G3P: glyceraldehyde 3-phosphate; DXP: 1-deoxy-D-xylulose-5P; GGDP: geranylgeranyl-2P; PDP: phytyl diphosphate; HGA: homogentisate; HPP: hydroxyphenylpyruvate; PEP: phosphoenolpyruvate; E4P: erythrose 4-phosphate; MPBQ: 2-methyl-6-phytylquinol; DMBQ: 2,3-dimethyl-5-phytylquinol; PP: phytyl phosphate.

Aiming to better understand the metabolic shift observed in *SlGLK2*-overexpressing line, a comprehensive transcript profile of key regulatory enzyme-encoding genes of the methylerythritol-phosphate pathway (*SlDXS* and *SlGGDR)*, shikimate pathway (*SlHPPD2*), tocopherol biosynthesis (*SlVTE1*, *SlVTE2*, *SlVTE3* and *SlVTE4*), and chlorophyll synthesis (*SlCHLG)*, chlorophyll dephytylation (*SlPPH* and *SlPPHL1*) and phytol recycling (*SlVTE5* and *SlVTE6)* were performed in green and ripe fruits (Fig 8, S6 Table).

Interestingly, an overall up-regulation of these genes was detected in L2 compared to control *au-Slglk2* in at least one of the analysed stages. The only exception was *SlHPPD2* gene, which was downregulated in *au-Slglk2* and L2 transgenic line compared to *SlGLK2* wild-type genotype. It is worth mentioning that all enzyme-encoding genes that were upregulated in *SlGLK2* genotype compared to *Slglk2* mutant shown in Fig 4 were also upregulated in L2 line, suggesting that they are indeed directly or indirectly regulated by SlGLK2 transcription factor.

In conclusion, SlGLK2 overexpression in *au-Slglk2* background led to the differentiation of more chloroplasts with highly-developed grana, which was accompanied by increased chlorophyll content in green fruits as well as by higher sugar accumulation and wild-type tocopherol levels in ripe fruits.

## Discussion

The crosstalk between light and phytohormones in tomato fruit chloroplast regulation has been increasingly investigated in recent years [6, 39, 50]. In this study, we focused our attention on the interplay between light, phytohormones and SlGLK2, as well as the signalling network of this tomato master transcription factor for fruit chloroplast differentiation.

The expression of *SlGLK2* is partially induced by light as it is diminished if green immature fruits develop in darkness [12]. However, the results presented here show that the *aurea* mutant had higher transcript amounts of *SlGLK2* in early stages of fruit development (Fig 1). The global deficiency in the PHY-mediated light perception characteristic of the *aurea* mutant has a much broader effect on plant physiology than solely depriving fruits of red/far-red light perception. For instance, such a deficiency has been shown to limit the auxin content and responsiveness in *aurea* immature fruits [39]. This not only explains the increment in *SlGLK2* transcripts (Fig 1), as auxin was found to transcriptionally repress this transcription factor as demonstrated in green fruits of the auxin resistant *dgt* mutant genotype (Fig 2), but also the anticipation of the *SlGLK2* mRNA peak in *aurea* fruits. This auxin-induced repression of *SlGLK2* might be mediated by the auxin-induced auxin-repressor *AUXIN RESPONSE FACTOR4* (*SlARF4)*. This transcription factor was found to downregulate *SlGLK1* expression in tomato fruits harbouring the mutated *Slglk2* allele [6], and putative ARF binding sites (TGTCTC box) can be recognized in *SlGLK2* promoter (S2A Fig). Moreover, both *aurea* [39] and *dgt* (S2B Fig) mutant genotypes display reduced levels of *SlARF4* transcripts at early stages of fruit development. Thus, it is not only the light signal that regulates *SlGLK2* expression, but also its interplay with auxin signalling.

Less clear is the relationship between cytokinins and *SlGLK2* expression. According to GUS activity assay, the cytokinin responsive *ARR5* promoter drove higher expression levels of *uid* gene in the presence of *SlGLK2* wild-type compared to mutant *Slglk2* allele in all fruit stages analysed (Fig 2). This result suggests that SlGLK2 positively regulates cytokinin signalling along fruit development and ripening. However, the constitutive overexpression of *AtCKX2*, which reduces the content of cytokinin, did not affect *SlGLK2* mRNA levels (Fig 2). This does not confirm the positive effect of cytokinins on *AtGLK2* expression described in *A*. *thaliana* root greening [3], supporting the existence of organ-specific regulatory networks.

To sum up, the PHY-mediated light regulation over SlGLK2 is most probably mediated by auxin through SlARF4, while SlGLK2 positively affects cytokinin signalling in fruits.

Fruits are generally regarded as photosynthate sinks that rely on energy provided by sugars transported from leaves to carry out the highly demanding processes of development and ripening [51]. However, in recent years, it has been increasingly demonstrated that tomato fruit plastid metabolism significantly influences nutritional and industrial quality of fruit [50]. Recent reports have shown that transcriptional factors enhancing chloroplast development in fruit may result in higher content not only of tomato fruit-specialized metabolites, *i*.*e*. carotenoids, but also of sugars [6, 12, 52]. SlGLK2 is within these transcription factors [13]; this study brings new, detailed data to this scenario.

The results obtained here by the analysis of the effect of the functional *SlGLK2* or the truncated protein-encoding *Slglk2* allele in wild-type or *aurea* mutant background provide further evidence regarding the role of this fruit specific transcription factor on nutritional quality, particularly on tocopherol content.

The presence of *SlGLK2* wild-type allele enhanced chlorophyll accumulation in immature fruits compared with those from *Slglk2* genotypes (Fig 3). This effect was accompanied by the enhancement of *de novo* synthesis of phytyl-2P (*SlDXS* and *SlGGDR*), chlorophyll biosynthesis (*SlCHLG*), and/or chlorophyll degradation and recycling (*SlPPH*, *SlPPHL1* and *SlVTE6*) (Fig 4). In *A*. *thaliana*, AtGLKs induce the expression of photosynthesis-related genes by the direct interaction with the promoter sequences of genes that function in light harvesting, such as LIGHT-HARVESTING CHLOROPHYLL-BINDING (AtLHCB) and key chlorophyll biosynthetic genes [3, 10]. Moreover, GLK proteins influence photosynthetic gene expression independently of the PHYs signaling pathway [10], in agreement with the results obtained for *SlGLK2*-overexpressing L2 plants in *aurea* background (Fig 6).

During tomato fruit ripening, *SlDXS* and *SlGGDR* expression is up- and downregulated, respectively; consequently, MEP pathways boost GGDP availability for carotenoid biosynthesis [27]. From breaker stage onwards, chlorophyll degradation-derived phytol is recycled to phytyl-2P, which feeds tocopherol biosynthesis [25]. Thus, there is a positive correlation between chlorophyll levels in green fruits and tocopherol amounts in ripe fruits [25]. In this regard, higher levels of tocopherol were observed in ripe fruits from *SlGLK2* than in *Slglk2*. Despite this, even with 3-fold more chlorophyll, ripe fruits from *SlGLK2*-overexpressing L2 plants in *aurea* mutant background displayed similar levels of tocopherol as wild-type fruits (Fig 5). Several reports demonstrated that phytyl-2P is the most limiting factor in tocopherol biosynthesis, both for *S*. *lycopersicum* [25] and *A*. *thaliana* [53]. However, this does not seem to be the case since phytol recycling is indeed enhanced by *SlGLK2* overexpression compared to *SlGLK2* wild-type ripe fruits by the upregulation of *SlVTE5* and *SlVTE6* expression (Fig 8). In this sense, tocopherol increment impairment might be the consequence of the shikimate precursor HGA limitation, reinforced by the fact that there was no effect of SlGLK2 on *SlHPPD2* transcript levels, with both *au-Slglk2* and L2 line showing decreased relative transcript abundance when compared to wild-type fruits (Fig 8). These results suggest that SlGLK2 positively regulates the tocopherol content in ripe fruits in two different ways: by increasing chlorophyll content during green stages of fruit development and by enhancing phytol recycling once ripening is triggered. Moreover, SlGLK2 participates in the regulation of chlorophyll, MEP and tocopherol core metabolic genes in a PHY-independent manner since the effect was observed in *aurea* mutant background.

Interestingly, when overexpressed in *aurea* background, *SlGLK2* reduced starch content in immature fruits and increased total soluble solids in ripe fruit (Fig 7), in part due to an increment in soluble sugars, similarly to what has been previously reported [12]. The combined effects of SlGLK2 and PHY-mediated light perception constitute an intricate regulatory network that controls carbon metabolism in a way yet to be revealed.

Recently, it has been shown that PHY-mediated light perception impairment reduced the mRNA levels of the auxin-induced *SlARF4* and enhanced the starch content in immature fruits [54]. Moreover, *SlARF4*-silenced tomato plants have higher expression and activity of AGPase, leading to an increment in the starch content observed in immature fruits from these plants [6]. Similarly, it has been demonstrated in cultured tobacco cells that auxins inhibit amyloplast development and transcriptionally repress starch biosynthesis-related genes [55]. Also, auxins, which are induced by PHY-mediated light perception [39], inhibit AMYLASE activity, delaying the accumulation of soluble sugars along ripening in climacteric fruits [56, 57]. In this context, given the fact that *SlGLK2* is downregulated by auxins and that SlGLK2 binding motifs [10] are found in *α-* and *β-AMYLASE* promotor sequence (S3 Fig), it is tempting to propose that *SlGLK2* mediates the auxin inhibition of *AMYLASE*. Altogether, SlGLK2 and PHY-mediated light perception affect soluble sugar content by controlling the balance between starch synthesis and degradation probably through the regulation of both AGPase and AMYLASE expression and/or enzyme activity.

**Fig 9:**
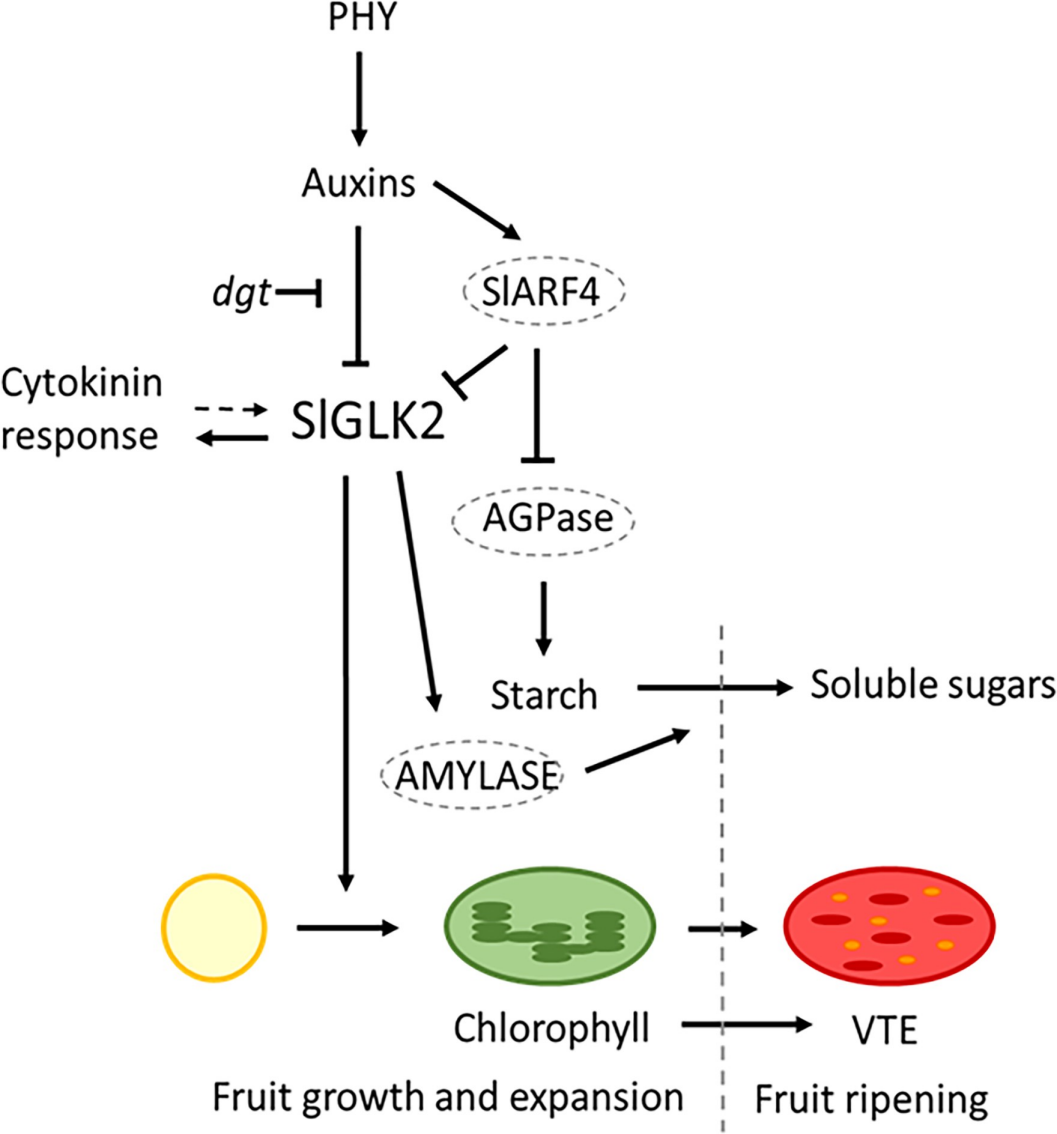
SlGLK2 signalling network. Phytochromes (PHY) acts as positive regulators of auxins [39], which, in turn, repress *SlGLK2* expression either directly or indirectly via SlAFR4, a negative regulator of auxin signalling [6]. In *dgt* background, an auxin response impaired genotype [33], the negative regulation of auxin over *SlGLK2* gene expression is disrupted. Cytokinin response is upregulated in the presence of SlGLK2. The positive effect of cytokinins over *AtGLK2* expression, described by [3] (dashed arrow), was not verified in tomato fruits. SlGLK2 is a master transcription factor that promotes the differentiation of proplastids into chloroplasts with the corresponding chlorophyll accumulation in green fruits, which is directly proportional to VTE content in ripe fruits. SlARF4 inhibits AGPase expression and enzyme activity [6]. Moreover, auxin is known to inhibit AMYLASE activity, which is associated with ripening-inducing starch degradation for soluble sugar accumulation in climacteric ripe fruits [56], probably mediated by SlGLK2. The balance between AGPase and AMYLASE enzyme activities determines the starch and, in part, soluble sugar content in green and ripe stages of fruits development.

In all, the results obtained here revealed that *SlGLK2* expression is negatively regulated by auxins in a phytochrome-dependent manner. By promoting the differentiation of proplastids into chloroplasts, SlGLK2 increases chlorophyll content at green stages of fruit development, which, during ripening, enhances phytol precursor availability for tocopherol biosynthesis. Additionally, by altering sugar metabolism, this protein also improves the total soluble solids of ripe fruits. In conclusion, SlGLK2 belongs to a complex signalling network that regulates chloroplast differentiation, maintenance and function, ultimately affecting the nutritional value of edible fruits. Although we cannot rule out that other unidentified variables and/or mechanisms could be underneath the observed responses, a tentative model for SlGLK2 signalling network can be drawn based on the experimental results obtained here as well as previously reported data (Fig 9). The complete understanding of all the factors involved and their relationships will be paramount to develop strategies to improve nutritional quality of tomato fruit.

## Supporting information

S1 Fig Phenotype of L6 T0 plant. (TIF)

S2 Fig *SlGLK2* is regulated by auxins. (TIF)

S3 Fig SlGLK2 binding motifs on *AMYLASE* promoter. (TIF)

S1 Table Primers used in the experiments. (XLSX)

S2 Table Relative transcript values of *SlGLK2* in tomato fruits from *SlGLK2*, *Slglk2* and *au-GLK2* genotypes. (XLSX)

S3 Table Tocopherol content in the pedicellar portion of fruits from *SlGLK2*, *au-SlGLK2* and *Slglk2* genotypes. (XLSX)

S4 Table Relative transcript values in the pedicellar portion of tomato fruits from *SlGLK2*, *Slglk2* and *au-GLK2* genotypes. (XLSX)

S5 Table Tocopherol content in the pedicellar portion of ripe fruits from *SlGLK2-*overexpressing L2 line. (XLSX)

S6 Table Relative transcript values in the pedicellar portion of tomato fruits from *SlGLK2*-overexpressing L2 line. (XLSX)
