# Supplementary figures and images for: Solanum lycopersicum GOLDEN 2-LIKE 2 transcription factor affects fruit quality in a light- and auxin-dependent manner
Source: PLoS One. 2019 Feb 12;14(2):e0212224. doi: 10.1371/journal.pone.0212224 (PMC6372215; doi:10.1371/journal.pone.0212224)

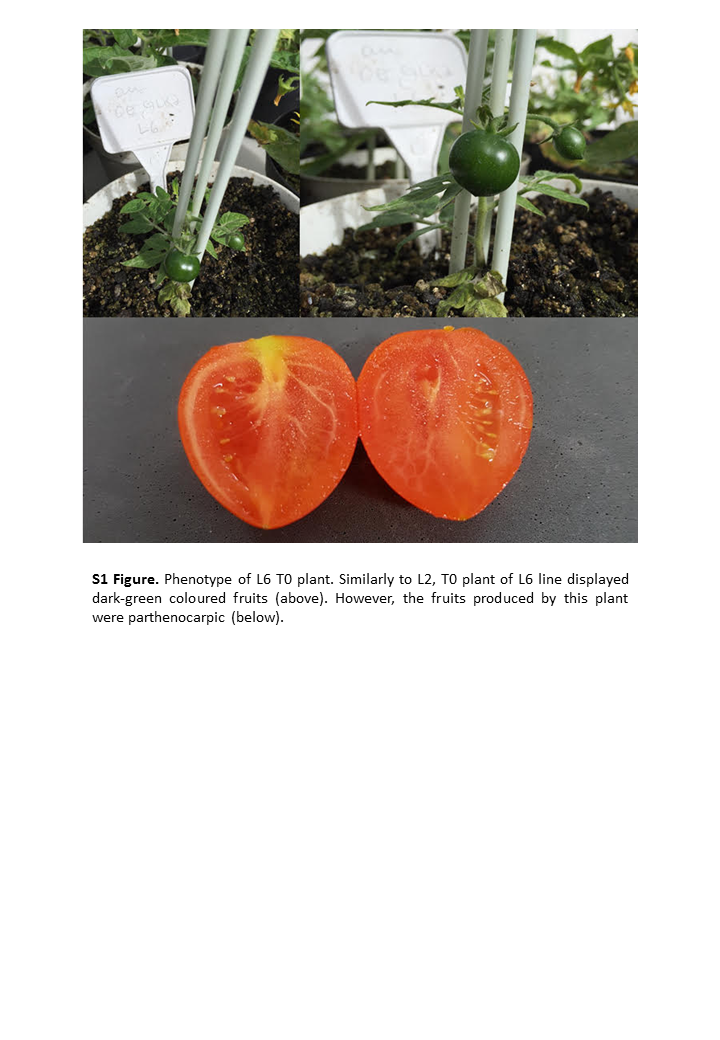

Supplement: S1 Fig — (TIF) [file pone.0212224.s001.tif]

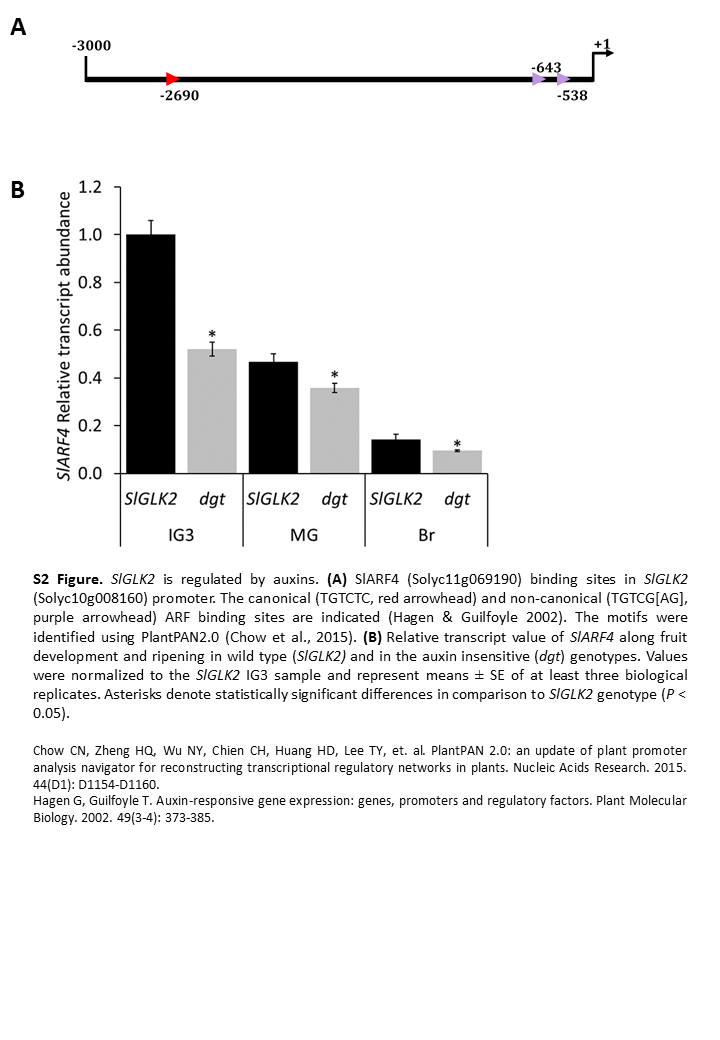

Supplement: S2 Fig — (TIF) [file pone.0212224.s002.tif]

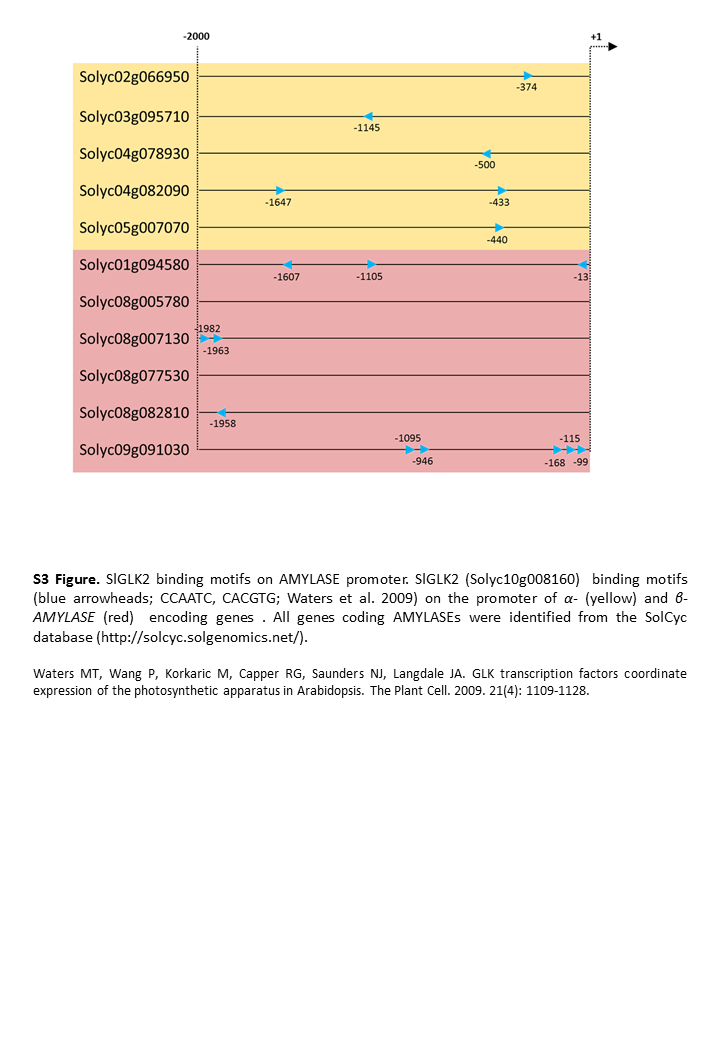

Supplement: S3 Fig — (TIF) [file pone.0212224.s003.tif]
